# Molecular-Orientation-Induced Rapid Roughening and Morphology Transition in Organic Semiconductor Thin-Film Growth

**DOI:** 10.1038/srep09441

**Published:** 2015-03-24

**Authors:** Junliang Yang, Sanggyu Yim, Tim S. Jones

**Affiliations:** 1 Institute of Super-microstructure and Ultrafast Process in Advanced Materials, School of Physics and Electronics, Central South University, Changsha, Hunan, 410083, China; 2 Department of Chemistry, Kookmin University, Seoul 136-702, Korea; 3 Department of Chemistry, University of Warwick, Coventry, CV4 7AL, United Kingdom

## Abstract

We study the roughening process and morphology transition of organic semiconductor thin film induced by molecular orientation in the model of molecular semiconductor copper hexadecafluorophthalocyanine (F_16_CuPc) using both experiment and simulation. The growth behaviour of F_16_CuPc thin film with the thickness, *D*, on SiO_2_ substrate takes on two processes divided by a critical thickness: (1) *D* ≤ 40 nm, F_16_CuPc thin films are composed of uniform caterpillar-like crystals. The kinetic roughening is confirmed during this growth, which is successfully analyzed by Kardar-Parisi-Zhang (KPZ) model with scaling exponents *α* = 0.71 ± 0.12, *β* = 0.36 ± 0.03, and 1/*z* = 0.39 ± 0.12; (2) *D* > 40 nm, nanobelt crystals are formed gradually on the caterpillar-like crystal surface and the film growth shows anomalous growth behaviour. These new growth behaviours with two processes result from the gradual change of molecular orientation and the formation of grain boundaries, which conversely induce new molecular orientation, rapid roughening process, and the formation of nanobelt crystals.

Organic electronics is an increasing attractive research field, especially with greatly potential commercialization of organic electronic devices such as organic light-emitting diodes (OLED), organic field-effect transistors (OFETs) and organic photovoltaics (OPVs)^1^,^2^,^3^,^4^. One of the key components in these devices is the active layer which is composed of organic semiconductor thin film. Controlling their morphology and structure can dramatically optimize the physical properties and accordingly improve the device performance^5^,^6^,^7^. The prerequisite for their controllability is to penetratingly understand the growth mechanism and evolution process of organic semiconductor thin film. Because of the inherent anisotropy of organic molecules, organic thin film growth shows more complex growth scenarios than conventional inorganic film. It normally exhibits its own growth features although sometimes the similar growth behaviour to inorganic thin film could be observed^8^,^9^,^10^,^11^,^12^,^13^,^14^,^15^,^16^,^17^. Furthermore, organic molecules with specific shapes, for example, rod-like molecule *para*-sexiphenyl (*p*-6P) and disk-like molecule metal phthalocyanine (MPc), often display different growth behaviours and mechanisms^11^,^18^,^19^.

In this study, we are dedicated to study the film roughening and morphology transition of organic molecular semiconductor in the model of copper hexadecafluorophthalocyanine (F_16_CuPc, Fig. 1a), which is an air-stable *n*-type material and widely used in OFETs and OPVs^20^,^21^. The experiments show that the growth of F_16_CuPc semiconductor thin film has two roughening processes with the thickness, *i.e*., traditional kinetic roughening and anomalously rapid roughening, accompanying morphology transition. The new growth mechanism supported by both experiments and simulation is unveiled that the gradual change of molecular orientation and grain boundaries results in the rapid roughening and morphology transition, and accordingly accelerate the formation of new molecular orientation and nanobelt crystals.

## Results and Discussion

The theoretical framework related to the mechanisms of film growth has been established in inorganic systems^22^, and was also developed to analyze the growth of organic thin films^9^,^14^,^17^,^19^. Height difference correlation function (HDCF) has been successfully analyzed the film morphological evolution, and the scaling exponents reflecting the film growth process and mechanism can be derived. The mean square height difference *g(R)* = <[h(x_1_, y_1_) − h(x_2_, y_2_)]> can be obtained based on all pairs of points (x_1_, y_1_) and (x_2_, y_2_) separated laterally by the distance *R* = [(x_1_ − x_2_)^2^ + (y_2_ − y_2_)^2^]^1/2^. The relative magnitudes of *R* and the correlation length, *ξ*, can divide the HDCF into two distinct behaviours: (1) *R* ≪ *ξ*, *g(R)* ∝ *R^2α^*, where *α* is the roughness scaling exponent; (2) *R* ≫ *ξ*, *g(R)* = *2σ*^2^, where *σ* is the surface root-mean-square (RMS) roughness. The parameters *ξ* and *σ* are dependent on the film thickness, *D*, and fit the power laws *σ* ∝ *D^β^* and *ξ* ∝ *D^1/z^*, where *β* and *z* are growth and dynamic scaling exponents, respectively. The ξ at each thickness can be determined by fitting the HDCF to the function *g(R)* = 2 *σ*^2^{1 − exp [−(*R*/*ξ*)*^2α^*]}. Based on the HDCF analysis, the scaling exponents *α*, *β*, and *z* can be obtained. Normally, they obey the scaling equation *β* ≈ *α/z*.

**Figure 1:**
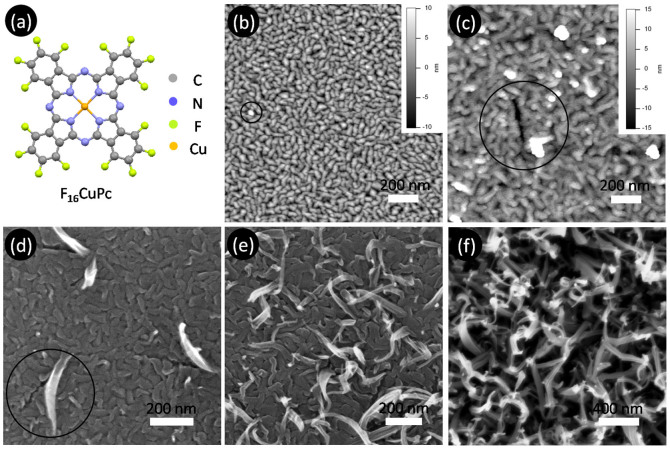
F_16_CuPc molecular structure (a) and film morphology (b–f) grown on SiO_2_ with different film thicknesses: (b) 30 nm, (c) 70 nm, (d) 70 nm, (e) 120 nm, (f) 260 nm. (b–c) are AFM images, and (d–f) are SEM images. The circles indicate the growth of nanobelt crystals on the caterpillar-like crystal layer.

Fig. 1 shows the morphology of F_16_CuPc films with different thicknesses grown on SiO_2_ substrate. The film morphology takes on an obvious transition from small caterpillar-like crystals to nanobelt crystals with increasing the thickness. As *D* < 30 nm, the film is composed of uniform caterpillar-like crystals. At *D* = 30 nm, some light spots can be found on the surface, as indicated by the circle in Fig. 1b. Increasing *D*, more and more light spots are formed (Fig. 1c). Furthermore, longer nanobelt crystals are also formed out of surface and leave a long gap. It suggests that the longer nanobelt crystals are lying-down on the surface at the beginning, and then they stand up when reaching a certain size for relaxing the strains (Fig. 1c and d). More and more nanobelt crystals can form accordingly as *D* continuously increases and finally form the fibre-like crystal surface (Fig. 1f). At *D* ≥ 70 nm, it is difficult to image the morphology of F_16_CuPc films with AFM because of longer standing-up nanobelt crystals, and SEM is used to image the films. It obviously indicates that thick films include two layers: caterpillar-like crystal layer and nanobelt crystal layer. The similar growth behaviour could also be found on the ITO substrate^23^.

**Figure 2:**
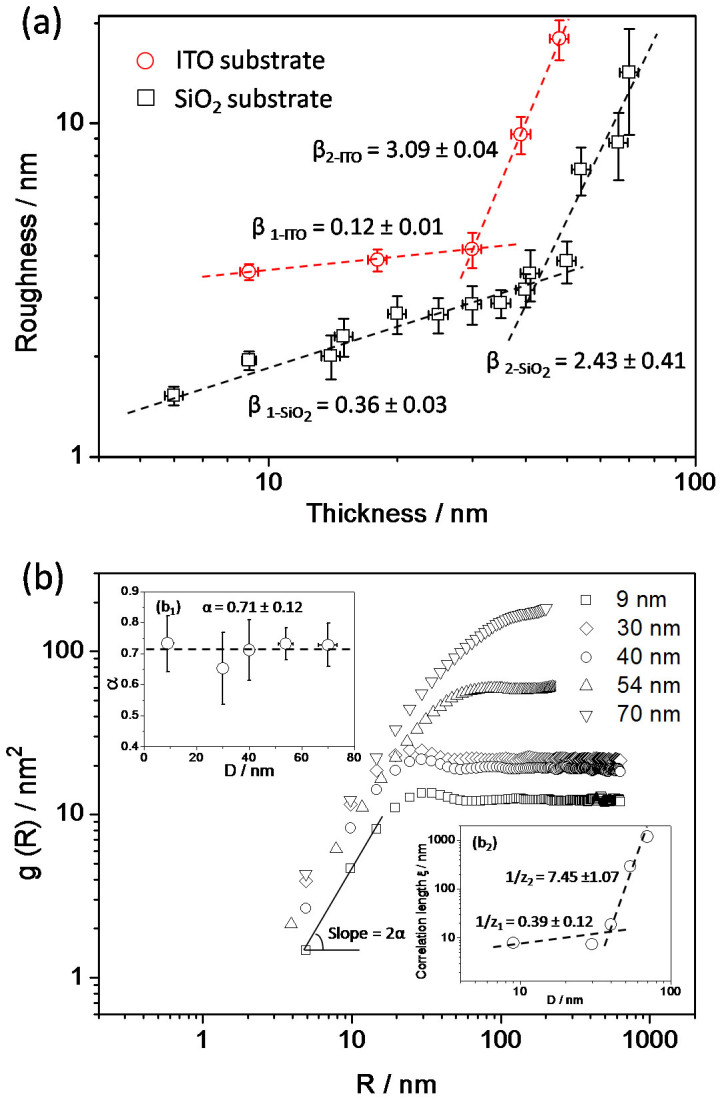
(a) Logarithmic plots of surface RMS roughness, σ, as a function of film thickness, *D*, for F_16_CuPc thin films. The data were obtained from AFM images. The σ values are the average values for scanning areas of 1, 2, and 5 μm^2^ on the ITO substrate^23^, and 1.5, 2.5, and 5 μm^2^ on the SiO_2_ substrate. (b) Logarithmic plots of averaged g(*R*) as a function of *R* with the film thickness *D* for F_16_CuPc thin films. The upper inset (*b_1_*) is the static roughness scaling exponent, *α*, as a function of D by fitting the linear part of g(R), where the average *α* is 0.71. The inset below (*b_2_*) is logarithmic plots of the correlation length, ξ, as a function of *D*, obtained by fitting HDCF to the analytical function g(R) = 2 σ{1 − exp[−(R/ξ)^2α^]}, where dynamic scaling exponents, *z*, can be obtained.

Based on the images by AFM (*D* ≤ 70 nm), the growth and roughening process can be analyzed. The surface RMS roughness, *σ*, as a function of film thickness, *D*, is shown in Fig. 2a. The data can be fitted using scaling equation *σ* ∝ *D^β^*. The growth-induced surface roughening normally includes kinetic roughening and mound growth. The absolute upper bound on the roughness fits the equation *σ* = *d*(*D*/*d*)^1/2^, where *d* is the molecular size, and *β* is usually 0.5. Analysis of the data for F_16_CuPc grown on SiO_2_ shows that *β* = 0.36 for *D* < 40 nm, while *β* = 2.43 for 40 nm ≤ *D* ≤ 70 nm (Fig. 2 also includes F_16_CuPc grown on the ITO substrate^23^, *β* = 0.12 for *D* ≤ 30 nm, while *β* = 3.09 for 30 nm ≤ *D* ≤ 48 nm. The growth of organic semiconductor thin film is related to the substrate, so the *β* and critical transition thickness show a little difference). These results suggest that F_16_CuPc film growth undertakes two different roughening mechanisms for *D* ≤ 40 nm and 40 nm ≤ *D* ≤ 70 nm on SiO_2_ substrate respectively. The dramatic increase in roughness for thick films can not be explained by conventional mound growth.

Fig. 2b shows the plot of the average HDCF, *g(R)*, as a function of film thickness, and an average value of *α* = 0.71 can be derived. On the other hand, the inverse dynamic scaling exponent 1/*z* is determined by logarithmic plots of the correlation length, *ξ*, as a function of *D*, with fitting HDCF to the analytical function *g(R)* = 2 *σ*{1 − exp[−(*R*/*ξ*)*^2α^*]}. It is obvious that two 1/*z* values, *i.e*., 1/*z*_1_ = 0.39 and 1/*z*_2_ = 7.45, are obtained divided at the critical thickness of 40 nm.

For *D* ≤ 40 nm, the growth behaviour can be analyzed with normal kinetic roughening and the scaling exponents are consistent with those based on Kardar-Parisi-Zhang (KPZ) model. In KPZ model, *α* ≈ 2/3, 1/*z* ≈ 0.3, and *β* ≈ 0.2; while in our case, *α* ≈ 0.71, 1/*z* ≈ 0.39, and *β* ≈ 0.36. The scaling exponents *α*, *β*, and *z* obey scaling equation 1/*z* ≈ *β*/*α*, so 1/*z*_cal_ ≈ 0.51, which is a little bigger than that obtained by experiment. This discrepancy results from the existence of different potential energy barrier at the step edges of existing islands^19^.

As the thickness increases and 40 nm ≤ *D* ≤ 70 nm (for *D* ≥ 70 nm, we cannot image the morphology of F_16_CuPc films with AFM because of longer nanobelt crystals and rough surface), the scaling exponents increase dramatically, in which 1/*z* ≈ 7.45 and *β* ≈ 2.43 although *α* is almost the same. The changes of scaling exponents are consistent with the transition of film morphology (Fig. 1b–d). This film growth behaviour can be divided into two roughening process: traditional kinetic roughening process and anomalous rapid roughening process accompanying morphology transition. In the later stage, the special large scaling exponents and growth behaviour cannot be analyzed with the existing mechanisms, *i.e*., kinetic roughening and mound growth, in the literatures. Here a new growth mechanism dominates the growth process.

**Figure 3:**
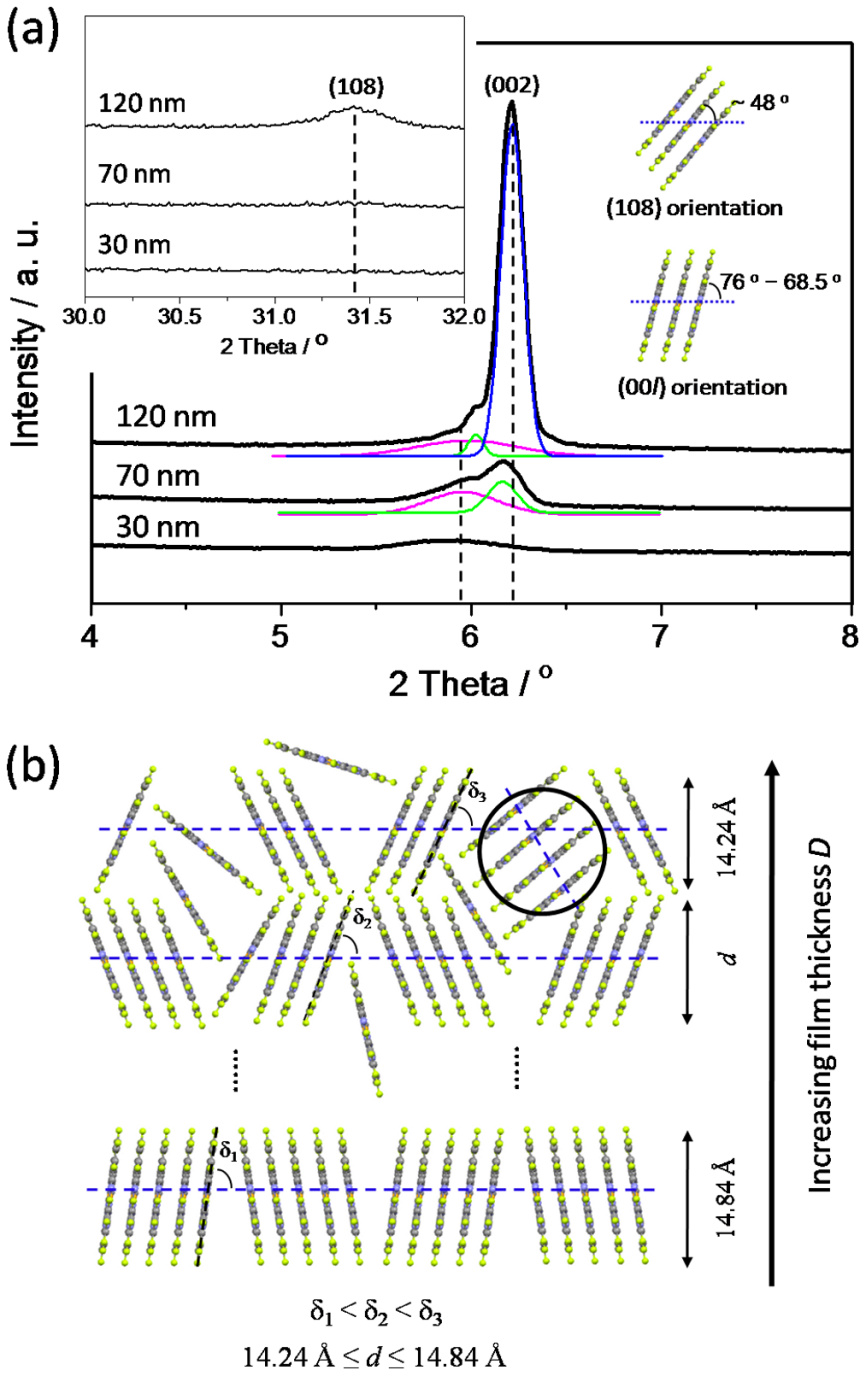
(a) XRD curves of F_16_CuPc films grown on SiO_2_ substrate with the thickness *D* = 30 nm, 70 nm and 120 nm, respectively. The main diffraction peaks show a gradual change in peak positions where new peaks at higher 2θ values emerge with increasing the film thickness. The fitted curves for the (002) diffraction peaks are shown as well. The left inset is the XRD data obtained between 30° and 32°. Increasing the film thickness not only changes the positions of (002) diffraction peak, but also a new diffraction peak emerges at 31.4°, indexed as (108). The right inset is the schematic of molecular arrangement of (00l) and (108) orientations. The diffraction peaks were indexed using single crystal data: *a* = 4.7960 Å, *b* = 10.228 Å, *c* = 28.002 Å, α = 86.41°, β = 87.89°, γ = 81.39° 24. (b) Schematic of F_16_CuPc molecular orientations in films as increasing the thickness *D*. The gaps between the grain boundaries or molecules become bigger as increasing the thickness *D*, in which it provides more probabilities that new molecules move into the gaps and arrange with new orientations. They aggregate, nucleate, grow up, and accelerate the formation of nanobelt crystals.

XRD experiments were carried out for these films. Fig. 3 shows the representative XRD curves of films with three different thicknesses. The main diffraction peaks show a gradual change in peak positions where new peaks at higher 2θ values emerge with increasing film thickness. The 30 nm film show only one diffraction peak at 2θ = 5.95° with *d* = 14.84 Å, indexed as (002). However, the 70 nm and 120 nm films obviously show one strong (002) diffraction peak with shoulders. For 120 nm films, the peak and shoulders can be fitted to three diffraction peaks, in which 2θ values are 5.95°, 6.02° and 6.20° respectively, with the *d* values can be obtained as 14.84 Å, 14.67 Å, and 14.24 Å accordingly. It suggests that molecular layer distance decrease slightly as increasing the thickness. Furthermore, a new peak emerges at 2θ = 31.4° with *d* = 2.84 Å for the 120 nm film, which can be indexed as (108) diffraction plane, as shown in the left inset in Figure 3a obtained between 30° and 32°. (This peak was not observed when F_16_CuPc films grown on ITO substrate^23^. It is because that F_16_CuPc film has a weaker crystallinity on ITO substrate than that on SiO_2_ substrate, and it is difficult to get the weak signal of (108) diffraction peak by normal XRD experiment.) It means, with increasing the thickness, not only the distance of molecular layer decreases, but also another molecular orientation emerges when the film thickness is over a critical value. The right inset in Fig. 3a shows the schematic of molecular arrangement of (00l) and (108) orientations.

Meanwhile, Fig. 3b shows the schematic of the change of molecular orientation with the film thickness *D*. The stacking angle δ decreases as the film thickness increases. For example, δ_1_ < δ_2_ < δ3, and it results in 14.24 Å ≤ *d* ≤ 14.84 Å when *D* ≤ 120 nm. The stacking angle δ decreases, the gaps between the grain boundaries or molecules increase. Thus it provides more probabilities that new molecules move into the gaps and arrange with new orientations. As indicated with the black circle in Fig. 3b, the (108) orientation forms in the gaps of (002) orientated crystals. After the nucleation happens with new molecular orientation, it can grow up and accelerate the formation of nanobelt crystals with new molecules coming under the continuous deposition. It is anomalous growth behaviour with rapid roughening process compared with the traditional KPZ film growth. Based on the experiment results, the critical thickness of the transition from KPZ to anomalous growth behaviour is about 40 nm for F_16_CuPc grown on SiO_2_. It suggests that no molecules or few molecules fill in the gaps of grain boundaries as D ≤ 40 nm, and it is advantageous to form smooth film with KPZ growth model. While D > 40 nm, the gaps become bigger and bigger with increasing *D*. Then more and more molecules move into the gaps with different orientations, in which they aggregate, nucleate, and grow up.

**Figure 4:**
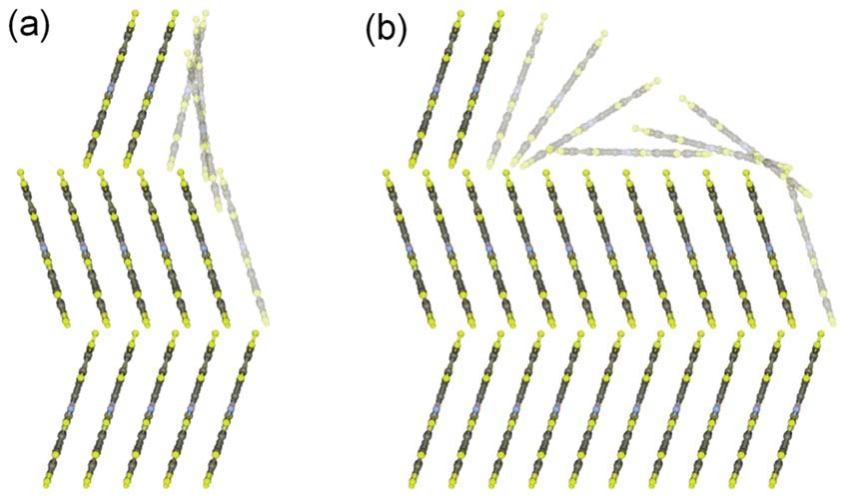
Molecular arrangements of (a) small and (b) large cluster models used for intermolecular interaction energy calculations. Impinging and then diffusing F_16_CuPc molecules are drawn palely.

In order to rationalize the scaling behaviour and roughening processes, the potential energy variation of impinging and then laterally diffusing F_16_CuPc molecules was simulated. Molecular structural parameters were obtained from density functional theory (DFT) methods, and the reported unit cell lattice parameters^24^ with *a* = 4.7960 Å, *b* = 10.228 Å, *c* = 28.002 Å, *α* = 86.41°, *β* = 87.89° and *γ* = 81.39° were used (The details are shown in the Supplementary Information). The intermolecular interaction energy calculation method and molecular mechanics force field parameters used were described elsewhere^25^,^26^. The calculations were performed for the two different crystal models consisting of three molecular layers as shown in Fig. 4. One model, denoted as Case A, with relatively narrow terrace (Fig. 4a) can represent the typical small clusters at the initial stage of the molecular deposition, *i.e.* *D* ≤ 40 nm. The other model, denoted as Case B, with relatively long terrace, can be a model for bigger clusters such as nanobelts of the thick films, *i.e.* *D* ≥ 70 nm. The intermolecular interaction energies of impinging F_16_CuPc molecule were then calculated as it diffuses laterally along *a* axis. In Fig. 4, the impinging molecules are drawn palely. The positions at *b* and *c* axes and molecular tilting values of the impinging molecule were varied to minimize the energy at each *a*-axis position.

**Figure 5:**
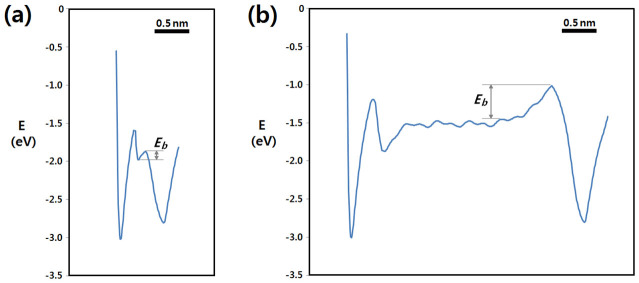
Intermolecular interaction energies for a diffusing F_16_CuPc molecule along a-axis calculated for (a) small and (b) large cluster modes.

The intermolecular interaction energies are plotted in Fig. 5. For both the models, the calculations yielded similar minimum energies of 3.01 eV at the energetically most stable position. However, it is interesting to note that significantly different energy barriers were observed at the step edges. The step edge barrier, *ΔE_b_*, at the largest value of 0.11 eV was observed for the Case A (Fig. 5a), while the *ΔE_b_* significantly increased to 0.44 eV for the Case B (Fig. 5b). The small step edge barrier for the Case A probably stemmed from nearly perpendicular molecular arrays. The impinging molecule moving at the step edge could easily slide down to the lower layer without breaking the intermolecular interactions a lot with existing molecules, as shown in Fig. 4a. This small edge barrier can explain the experimental results that the growth behaviour of F_16_CuPc thin films when *D* ≤ 40 nm was consistent with conventional scaling behaviour based on the KPZ model. In contrast, for the Case B, the impinging molecule on the large terrace was stabilized by adopting a configuration with its molecular plane almost parallel to the surface in order to maximize the intermolecular interactions, as shown in Fig. 4b. This configuration breaks at the step edge when the diffusing molecule moves down to the lower layer, leading to a significant decrease in the molecular interactions and large energy barriers. This kind of large step edge barrier is similar to the growth of PTCDA thin films with surface-parallel orientation^27^. The simulation supports the gradual change of molecular orientations and the formation of nanobelt crystals with increasing the film thickness.

## Conclusion

In conclusion, we use both experiment and simulation to study organic semiconductor thin-film growth in the model of molecular semiconductor F_16_CuPc. It is revealed that molecular semiconductor thin-film growth can take on more than one processes, *i.e*., conventional KPZ growth and anomalous growth behaviour with rapid roughening process, accompanying morphology transition as the film thickness increases. This new growth behaviour results from the gradual change of molecular orientation and the formation of grain boundaries, which induce new molecular orientations, rapid roughening process, and the formation of nanobelt crystals.

## Methods

### Materials and Thin Film Growth

F_16_CuPc material (Sigma-Aldrich) was purified twice by thermal gradient sublimation prior to use. Films with different thicknesses were grown by high vacuum organic molecular beam deposition (base pressure of about 4 × 10^−8^ mbar) with the substrate held at room temperature. All films were grown on either commercially available pre-cleaned SiO_2_ (IDB Technologies Ltd) or indium tin oxide (ITO)-coated glass (CRL Opto) substrates with root-mean square (RMS) roughness of ~0.4 nm and ~3.5 nm respectively. A deposition rate of 0.15–0.20 Å s^−1^ was used for all F_16_CuPc thin films.

### Characterization

The morphology of fresh films was characterized by AFM (Asylum Research MFP-3D, Santa Barbara, USA) with taping mode and field emission SEM (FE-SEM, Zeiss Supra 55VP, Germany) with operating voltage at 5 kV. X-ray diffraction (XRD) was used to characterise the structure of films using an X′ Pert PRO (PANalytical, Netherlands) instrument with Cu Kα radiation (λ = 1.540 56 Å), where the selected voltage and current were 45 kV and 40 mA, respectively. The scanning rate was set at 0.4°/min from 2° to 32° (2*θ*).

## Author Contributions

J.L.Y. and T.S.J. conceived the idea. J.L.Y. carried out all the experiments as well as the data collection and analysis. S.Y. contributed to the simulation calculation and analysis. J.L.Y. and S.Y. prepared the first draft. All authors discussed the results and provided comments on the manuscript.

## Supplementary Material

Supplementary Information Supplementary Information
