# Supplementary material for: Molecular-Orientation-Induced Rapid Roughening and Morphology Transition in Organic Semiconductor Thin-Film Growth
Source: Sci Rep. 2015 Mar 24;5:9441. doi: 10.1038/srep09441 (PMC4371086; doi:10.1038/srep09441)
Supplement: Supplementary Information [file srep09441-s1.doc]

# Supplementary Information

# Molecular-Orientation-Induced Rapid Roughening and

# Morphology Transition in Organic Semiconductor Thin-Film Growth

Junliang Yang, ^† *^ Sanggyu Yim, ^‡^ Tim S. Jones ^§ *^

*^†^ Institute of Super-microstructure and Ultrafast Process in Advanced Materials, School of Physics and Electronics, Central South University, Changsha, Hunan, 410083, China*

*^‡^ Department of Chemistry, Kookmin University, Seoul 136-702, Korea*

*^§^ Department of Chemistry, University of Warwick, Coventry, CV4 7AL, United Kingdom*

* Corresponding author. [Junliang.yang@csu.edu.cn](mailto:Junliang.yang@csu.edu.cn); Tel: +86-731-88660256

[T.S.Jones@warwick.ac.uk](mailto:T.S.Jones@warwick.ac.uk); Tel: +44-24-76528265

**Molecular orientation in F_16_CuPc unit cell**


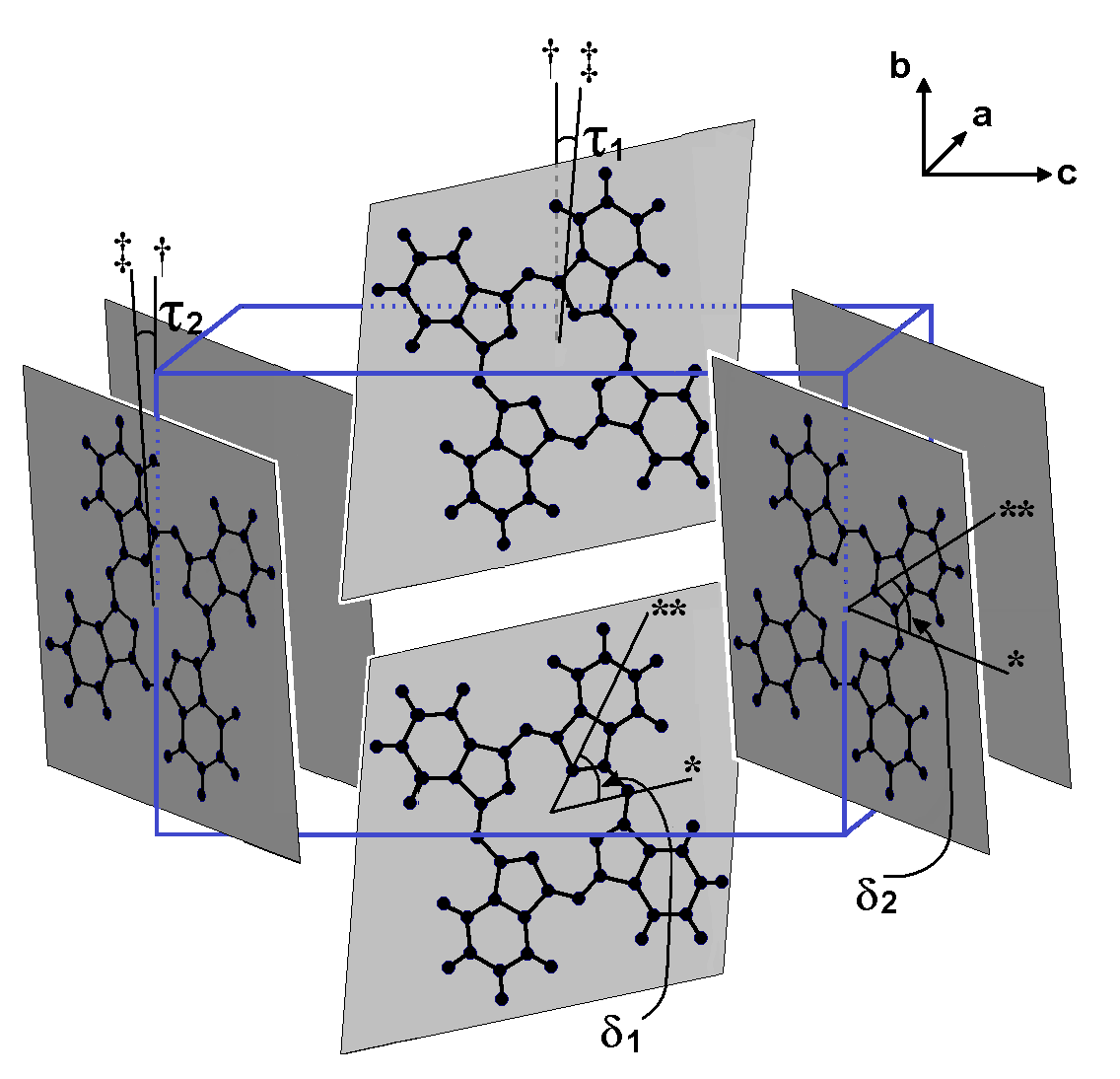


**Fig. S1** Structural model for F_16_CuPc unit cell. τ_1_ and τ_2_ are angles between †, the direction normal to the a-c plane and ‡, the direction of the molecular plane. δ_1_ and δ_2_ are the angles between *, the intersection of the molecular plane and the a-c place, and **, the molecular axis defined as the line passing through the two opposite pyrrole nitrogens.

The intermolecular interaction energy of the F_16_CuPc unit cell (Fig. S1) was calculated by varying four angle parameters, two tilt angles (τ_1_ and τ_2_) and two rotation angles (δ_1_ and δ_2_), using molecular mechanics (MM) method. The definition of the angles was described elsewhere [1]. The fixed unit cell lattice dimensions were *a* = 4.7960 Å, *b* = 10.228 Å, *c* = 28.002 Å, *α* = 86.41°, *β* = 87.89° and *γ* = 81.39° [2]. At specific parameters, the intermolecular interaction energy of the unit cell is the sum of the Lennard-Jones vdW energies for all possible atom pairs.


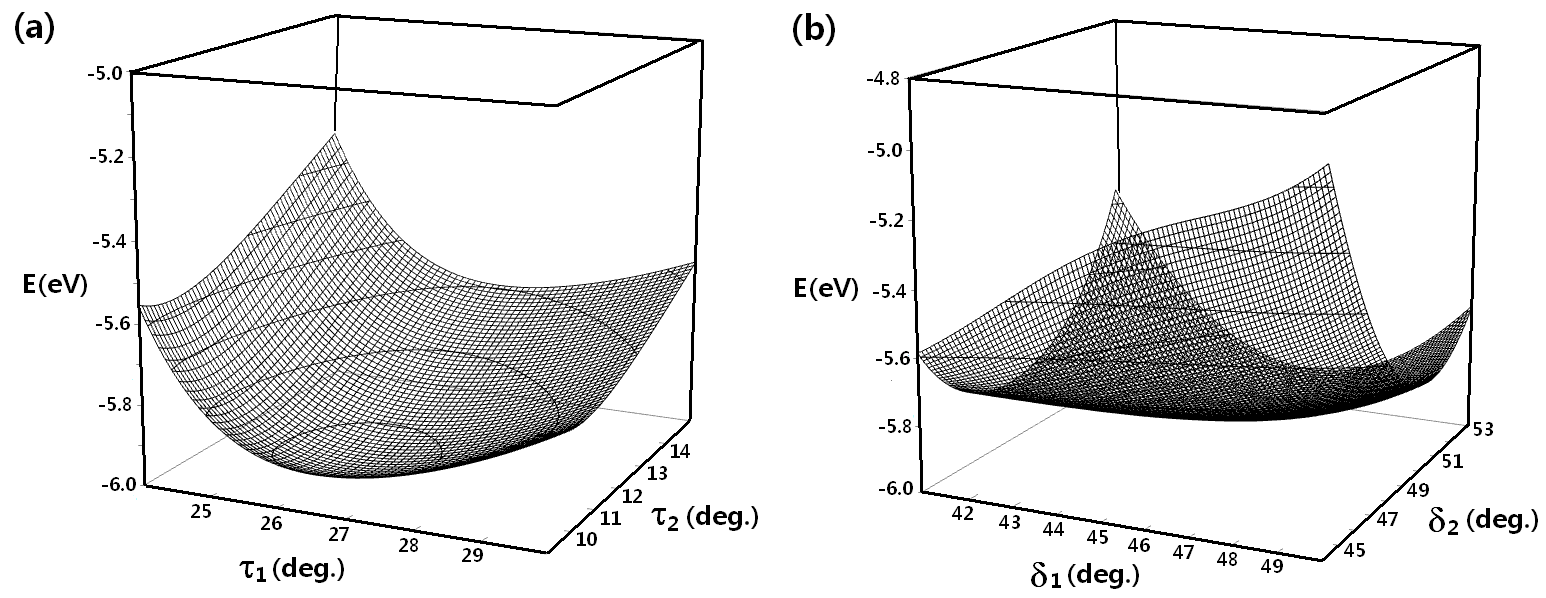


**Fig. S2** Intermolecular interaction energy surface diagrams for the F_16_CuPc unit cell as a function of (a) τ_1_ and τ_2_ at δ_1_ = 45.5° and δ_2_ = 50.7°, and (b) δ_1_ and δ_2_ at τ_1_ = 26.6° and τ_2_ = 10.0°.

The unit cell has a minimum energy of -5.93 eV at τ_1_ = 26.6°, τ_2_ = 10.0°, δ_1_ = 45.5° and δ_2_ = 50.7°. The energy per molecule corresponds to -2.97 eV since the unit cell consists of two molecules. The energy surfaces as a function of angle parameters are shown in Fig. S2. Fig. S2a shows the energy surface drawn with respect to the molecular plane tilt angles, τ_1_ and τ_2_, when the in-plane molecular rotation angles, δ_1_ and δ_2_, are fixed to 45.5° and 50.7°, respectively, and Fig. S2b shows the energy surface drawn with respect to δ_1_ and δ_2_ with the tilt angle values of τ_1_ = 26.6° and τ_2_ = 10.0°.

**References**

[1] S. Yim, S. Heutz, T. S. Jones, *Phys. Rev. B*, 2003, 67, 165308.

[2] S. M. Yoon, H. H. Song, I. C. Hwang, K. S. Kim and H. C. Choi, *Chem. Commun.*, 2010, 46, 231.
